# Trophic state resilience to hurricane disturbance of Lake Yojoa, Honduras

**DOI:** 10.1038/s41598-023-32712-3

**Published:** 2023-04-07

**Authors:** J. M. Fadum, M. N. Waters, E. K. Hall

**Affiliations:** 1 Graduate Degree Program in Ecology, Colorado State University, Fort Collins, CO 80523 USA; 2 Department of Ecosystem Science and Sustainability, Colorado State University, Fort Collins, CO 80523 USA; 3 Department of Crop, Soil and Environmental Sciences, Auburn University, Auburn, AL 36849 USA

**Keywords:** Freshwater ecology, Limnology

## Abstract

Cyclones are a poorly described disturbance in tropical lakes, with the potential to alter ecosystems and compromise the services they provide. In November 2020, Hurricanes Eta and Iota made landfall near the Nicaragua-Honduras border, inundating the region with a large amount of late-season precipitation. To understand the impact of these storms on Lake Yojoa, Honduras, we compared 2020 and 2021 conditions using continuous (every 16 days) data collected from five pelagic locations. The storms resulted in increased Secchi depth and decreased algal abundance in December 2020, and January and February 2021, and lower-than-average accumulation of hypolimnetic nutrients from the onset of stratification (April 2021) until mixus in November 2021. Despite the reduced hypolimnetic nutrient concentrations, epilimnetic nutrient concentrations returned to (and in some cases exceeded) pre-hurricane levels following annual water column turnover in 2021. This response suggests that Lake Yojoa’s trophic state had only an ephemeral response to the disturbance imposed by the two hurricanes, likely due to internal input of sediment derived nutrients. These aseasonal storms acted as a large-scale experiment that resulted in nutrient dilution and demonstrated the resilience of Lake Yojoa’s trophic state to temporary nutrient reductions.

## Introduction

Large lakes across the world are key resources for the persistence and subsistence of human and wildlife populations, providing refugia for biodiversity, cultural significance to communities, and fuel for local economies^1^. However, local anthropogenic stressors and global changes, such as shifting climate patterns, threaten the ecosystem services supported by these lakes, and by extension, the communities who depend on them^2^. One such shifting climate pattern is the rise of extreme storm events such as increasing tropical cyclone frequency and intensity on account of rising sea surface temperatures^3,4^. Lakes and communities in Central America are particularly threatened by intensifying cyclones that develop in the Atlantic basin. In addition, Central American communities may be especially vulnerable to cyclone-driven shifts in ecosystem services due to a dependence of regional economies on agriculture and hydropower^5^. Regional climate projections suggest that Central America is likely to experience a drier future climate^5,6^, with a higher frequency of more intense storms over the coming decades^7^. As such, the equitable and sustainable management of aquatic resources under future climate scenarios rests largely on maximizing resiliency through developing an understanding of how present weather events influence the ecology of impacted ecosystems.

The degree to which a particular tropical cyclone impacts an aquatic ecosystem depends on individual storm characteristics such as wind speed, amount of rainfall, and the specific path of the cyclone^8^. Some tropical ecosystems may be resistant or resilient to storm disturbance^9^. Alternatively, because of the novelty of current and predicted storm regimes, lakes are also likely to experience altered thermal stability^10^, increased suspended sediment concentrations with resulting changes in light availability^11^, and increased delivery of allochthonous materials that fuel primary productivity^12^. These combined impacts can dramatically alter ecological function^13^, particularly in shallow lakes^14,15^. Though larger, inland lakes may be more resilient to the immediate impacts of storm events than polymictic and coastal lakes, inland lakes are still subjected to extreme precipitation events related to tropical cyclones. These precipitation extremes are particularly relevant in tropical aquatic ecosystems due to the relationship between seasonal precipitation and the delivery of watershed derived nutrients. The timing of precipitation driven nutrient additions interfaces with thermo-physical water column structure and stratification phenology to influence annual nutrient dynamics which determine trophic state. Thus, cyclone driven alterations to the interactions between nutrient loading and thermo-physical structure may have measurable consequences to the trophic state of tropical lakes. Furthermore, determining if tropical cyclones cause episodic limnological changes or permanent shifts in trophic state is critical to evaluating ecological resiliency to storms.

**Figure 1:**
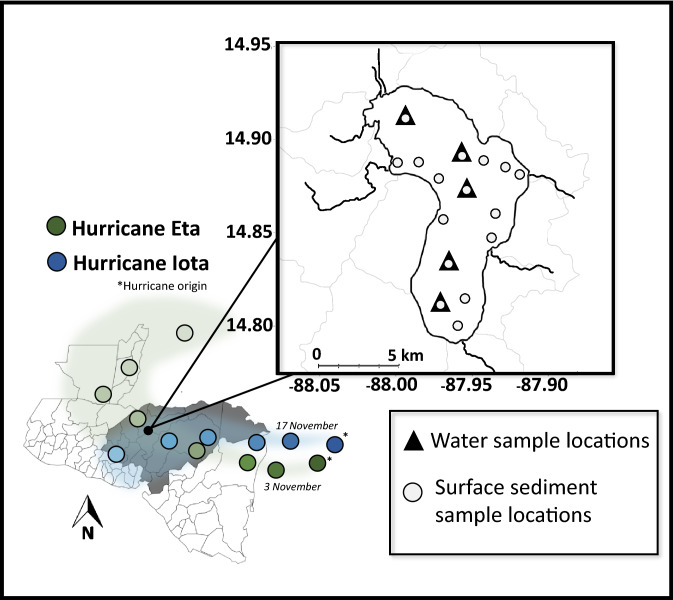
Sampling locations (water column and surface sediments) in Lake Yojoa and approximate paths of Hurricanes Eta and Iota. Color gradation (dark to light) denotes direction of the cyclones. Map outlines generated using MapChart (www.mapchart.net, 2022; Creative Commons Attribution-ShareAlike 4.0 International License). Paths based on National Hurricane Center assessments^16,17^.

In November 2020, Hurricanes Eta and Iota crossed over Lake Yojoa, Honduras during a period of continuous data collection, providing an opportunity to measure whole ecosystem responses to these anomalous but increasingly frequent precipitation events. The Lake Yojoa watershed experienced its first massive late-season precipitation event of 2020 from November 3–5 when Hurricane Eta (Saffir-Simpson Hurricane Wind Scale: Category 4) made landfall near the Nicaragua-Honduras border before dissipating to a tropical depression (Fig. 1)^16^. This initial storm event was followed only 12 days later by the second hurricane (Hurricane Iota) which made landfall in a nearly identical location in eastern Nicaragua on November 17 (Fig. 1)^17^. Shortly before making landfall, Hurricane Iota was classified as a Category 5 storm, making it the latest date a Category 5 hurricane had developed in the Atlantic basin since hurricane records began. Hurricane Iota weakened to a tropical depression on November 18 as it moved towards the Honduras-El Salvador border, but not before generating a second immense precipitation event that brought additional rainfall to Honduras’s already saturated ecosystems, including Lake Yojoa. While exact precipitation values are unavailable because the storms overwhelmed local rain gauges, best estimates are that the storms brought over 350 mm of rain to the Lake Yojoa watershed, with surrounding areas receiving nearly a meter of rainfall^16,17^. The year prior (2019), the Lake Yojoa watershed received 99.6 mm of rainfall in November, typical for the area. Thus, each storm produced as much precipitation within the span of a few days as the watershed might otherwise expect to see in the entire month. Hurricanes Eta and Iota exceeded the precipitation produced by cyclones in recent years but did not inflict nearly as much damage as Hurricane Mitch in 1998, which produced massive flooding and resulted in 5,657 documented fatalities^18^.

In recent decades, Lake Yojoa has transitioned from an oligotrophic to mesotrophic ecosystem with an annual mean Secchi depth of just over 3 m (compared to over 7 m in the 1980s)^19^. One key mechanism for maintaining Lake Yojoa’s recently elevated trophic state is intra-annual hypolimnetic nutrient accumulation during seasonal stratification (typically April to November) combined with nutrient release to the photic zone during annual mixing^19^. Therefore, changes in nutrient concentrations in both surface and deep waters is a critical metric for understanding trophic state development in this ecosystem. We hypothesized that Hurricanes Eta and Iota could have two possible effects on Lake Yojoa; a nutrient loading effect, wherein a large amount of terrestrial nutrients would be transported to Lake Yojoa by surface run-off from the storms and/or a diluting effect where the large quantity of nutrient-deplete runoff would decrease pelagic nutrient concentrations. It is also possible that each storm may have had a different effect on Lake Yojoa. To test for these hypothesized changes following the hurricanes, we compared Secchi depth and concentrations of chlorophyll* a* (Chl-a), NH_4_^+^, NO_3_^−^, total phosphorus (TP), dissolved organic carbon (DOC), and particulate carbon (C) and particulate nitrogen (N) in surface (1 m) and deep (16 m) water in 2020 (before the hurricanes) and 2021 (after the hurricanes). The sampling covered seasons when the water column was fully mixed and when the lake was stratified, both before and after the hurricanes.

## Methods

### Lake Yojoa

Lake Yojoa is the largest natural freshwater lake in Honduras (~ 83 km^2^ surface area, 1.4 km^3^ volume, annual maximum depth between 24.6 and 29 m, approximate) and is a critical economic and water resource in the region. With an area of ~ 337 km^2^, the Lake Yojoa watershed supports three municipalities (Las Vegas, La Guama, and Peña Blanca), two protected national parks (Parque Nacional Cerro Azul and Parque Nacional Montaña de Santa Bárbara) and is a popular destination for domestic tourism, with a suite of hotels and restaurants that border the lake. The watershed experiences persistently warm temperatures (annual mean air temperature = 23.4 C in 2019), with the warmest months corresponding with the monsoon season. The majority of precipitation (~ 85%) falls between June and October (based on 2019 measurements) and overlaps with Lake Yojoa’s stratified water column months (typically April to November)^19^. An industrial net-pen Tilapia aquaculture operation, located in the northern region of the lake, likely contributes large quantities of nutrients to Lake Yojoa^19^, with additional allochthonous nutrients being delivered during the monsoon season. El Mochito mine, located in the western region of the watershed has contributed heavy metals to Lake Yojoa’s sediments since production began in 1948^20^. Previous estimates of land use and land cover (LULC) suggest that the watershed is approximately 45% forested (largely included within the two national parks), 20% traditional agriculture dominated by coffee, beans, and corn, 15% modern agriculture dominated by coffee and sugar cane production, 10% pasture or grassland and less than 5% urban or wetland^21^. However, a contemporary assessment of LULC is unavailable.

### Sampling

We collected water for Chl-a and nutrient analyses (NH_4_^+^, NO_3_^−^, TP, DOC, and particulate C and N) from five stations (Fig. 1) at two depths (1 and 16 m) using an opaque Van Dorn water sampler every 16 days from January 2020 through December 2021 (excepting April 2020 due to COVID-19 restrictions). All five stations are located in the pelagic zone of Lake Yojoa and range in depth (across sites and across seasons) from 18 to 29 m (Fig. 1). During each sampling event, at each station we recorded Secchi depth, and temperature and dissolved oxygen (DO) profiles at two-meter intervals using a Hydrolab MS5 Multiparameter Sonde (OTT HydroMet, Loveland, CO). Water (from 1 and 16 m) was stored in opaque HDPE Nalgene bottles and immediately placed in a cooler with ice packs until we returned to the laboratory (approximately 1–2 h after sampling). Once in the laboratory, whole water samples were frozen for TP analysis while NH_4_^+^, NO_3_^−^, and DOC samples were first filtered through 25 mm glass microfiber filters, Grade GF/F (pore size = 0.7 µm) via Polysulfone filter funnels, and subsequently frozen. Due to the time elapsed between collection, filtering, and freezing, the reactive nutrients measured in the collected samples reflect the net balance of assimilation and recycling processes occurring in water samples between collection and analyses, not necessarily exact in situ concentrations. However, transporting unfiltered water samples in a dark cooler with ice packs and freezing samples immediately after filtration likely reduced any unquantified differences between in situ and measured concentrations to the fullest extent possible given logistical constraints associated with sampling.

Filters from 1 m were analyzed for Chl-a and filters from 16 m were analyzed for particulate C and N. All filters were folded in half, wrapped in aluminum foil, and immediately frozen. Water samples, stored in 15 ml centrifuge tubes, and filters were transported to Fort Collins, CO, USA while frozen. Nutrient analyses were performed at the EcoCore facility at Colorado State University, Fort Collins, CO, USA as described below.

In addition, we also collected surface sediments using an Eckman dredge in January 2020, prior to hurricane activity. Sediments were collected at 16 sites (Fig. 1) throughout the lake, including locations in the deepest regions of the lake where the water column fully stratifies as well as locations near the large net-pen aquaculture operation in the northern basin, and nearer to tributary inputs which differed in land use inputs (e.g., mining, restaurants, protected areas). Sediment samples were freeze-dried and ground with a mortar and pestle prior to analysis.

### Laboratory and statistical analyses

Lab analyses were consistent with previous research we have conducted for Lake Yojoa^19^. Briefly, Chl-a was measured on a 10-AU fluorometer (Turner Designs Part No. 10-AU-074, calibrated using Turner Designs liquid Chl-a standards) via extraction in 90% acetone with acidification to 0.003 N HCl with 0.1 N HCl^22^. TP was first digested to soluble reactive phosphorus (SRP) via the Alkaline Potassium Persulfate method under sub-boiling (90 C) temperature, then measured using a colorimetric ascorbic acid assay (EPA Method 365.3) modified to be analyzed on a UV-STAR 96-well Microplate via Infinite M200 TECAN at 880 nm absorbance detection. NH_4_^+^ and NO_3_^−^ were measured using a Flow Solution FS 3700 Automated Chemistry Analyzer (O.I. Analytical, College Station, TX). DOC was measured on a Shimadzu TOC-L (Shimadzu Scientific Instruments, Inc). Particulate C and N were measured on a Velp 802 elemental analyzer.

Sedimentary organic C and N were measured using a Costech Elemental CHN analyzer and reported as percentages. Prior to analysis, sediment samples were fumigated in HCl vapors for 24 h to remove carbonates. Sedimentary P was measured using an ICP analyzer following digestion with 15N HNO_3_ in a heated block following EPA method 6010B. P is reported as mg g^−1^ of sediment.

Statistical analyses were preformed, and figures were created in R (version 4.1.2) using packages *tidyverse*^23^, *tidyr*^24^, *lubridate*^25^, and *ggeasy*^26^. Comparisons (monthly and annual) between pre-hurricane and post-hurricane parameters were evaluated using one-way analysis of variance (ANOVA). “Pre-hurricane” conditions refer to observations from January-December 2020 whereas “post-hurricane” conditions refer to observations from January-December 2021. To ensure that observations in November and December 2020 (the weeks following the hurricanes) did not impact results, we also compared observations from January 2020 to October 2020 to those from November 2020 to December 2021. The two temporal groupings did not affect the statistical significance of differences of annual means and our conclusions were unaffected by the choice of comparison grouping. Therefore, all comparisons were grouped by calendar year for simplicity.

## Results

**Figure 2:**
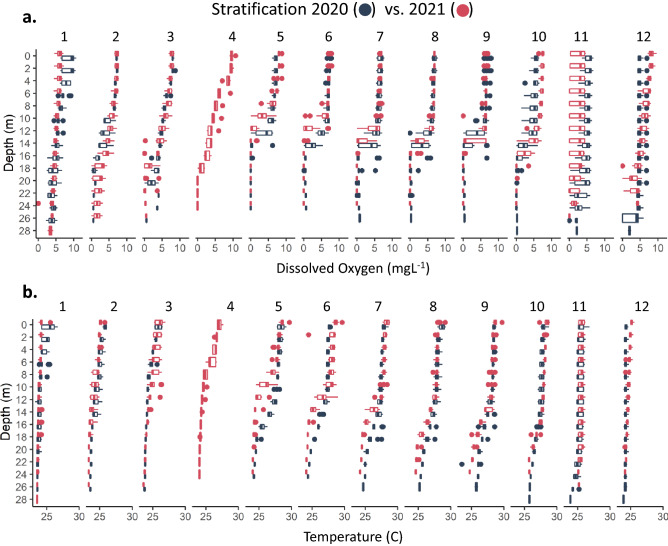
Profiles of monthly. (**a**) Dissolved oxygen and (**b**) temperature (minimum, maximum, inter-quartile range with outliers at 2 m intervals).

To understand the impacts of Hurricanes Eta and Iota, we assessed trophic state before and after the hurricanes using Secchi depth and Chl-a concentrations and paired these observations with measurements of nutrient concentrations and thermo-physical structure. To evaluate potential drivers of changing trophic state, we began by identifying the timing of water column mixus in 2020. The Lake Yojoa water column mixed between October 22 and November 7 in 2020 (Fig. 2) and again in late October 2021. The timing of mixing in 2020 and 2021 was consistent with observations from previous years, including 2019 when the water column mixed between October 28 and November 13^19^. While we were unable to determine if the 2020 mixus was initiated by Hurricane Eta, as hurricanes are capable of mechanically mixing water columns^27^, the consistency of the timing of 2020 mixus with previous years suggests that observed changes in thermo-physical structure and nutrient dynamics in 2021 can be attributed to Hurricanes Eta and Iota, and not to premature water column destabilization or early hypolimnetic nutrient release.

**Figure 3:**
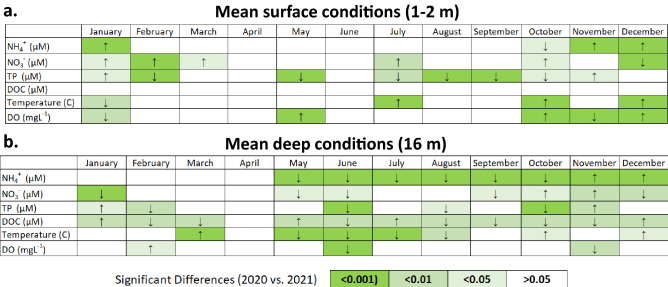
Statistical significance of differences in mean monthly nutrient concentrations at 1 m, and temperature and dissolved oxygen at (**a**) 1–2 m (2020 vs. 2021) and (**b**) 16 m. Arrows indicate direction of change (2020 to 2021) in months where *p* < 0.05 (i.e., ↑ indicates an increase in 2021 compared to 2020 whereas ↓ indicates a decrease in 2021 compared to 2020). Cell color denotes *p* value (2020 vs. 2021, ANOVA).

In the two years on either side of the hurricanes (2020 vs. 2021 calendar years), we observed no difference in annual mean DO in surface (depth = 2 m) waters (*p* = 0.19, annual mean ± standard error (SE), 2020 = 6.50 ± 0.14 mgL^−1^, 2021 = 6.80 ± 0.17 mgL^−1^; Supplementary Table S1). While there was a difference between the two years in surface DO concentration for five of the eleven months we compared (January, May, October, November, and December, Fig. 3a), there was no consistent directional change, with concentrations of DO in some months in 2021 being below 2020 values and others being above 2020 values (Supplementary Table S1; Fig. 3a). Similarly, there was no difference in annual mean DO in deep (depth = 16 m) waters (*p* = 0.31, annual mean ± SE, 2020 = 2.35 ± 0.25 mgL^−1^, 2021 = 2.02 ± 0.20 mgL^−1^; Supplementary Table S1). DO in deep water was only different in three months (February, June, and November, Fig. 3b), with no directional change.

These inconsequential changes in DO at 2 m and 16 m corresponded with the oxycline depth being unchanged between years. In 2020, the fully developed oxycline was, on average, at 13.0 m, compared to 13.7 m in 2021 (Fig. 2). Therefore, whereas there were clear differences in DO between years at certain depths and for certain months (Figs. 2a,b, 3), the lack of consistent directional change suggests that these differences could not be attributed to the storm events. In both 2020 and 2021, stratification driven hypoxia was fully established by May and water column mixus and reoxygenation of the full water column occurred in late October or early November (Fig. 2).

Similar to DO, annual mean surface water temperature was not different between pre (2020) and post (2021) hurricanes (*p* = 0.92, annual mean ± SE, 2020 = 26.71 ± 0.16 °C, 2021 = 26.69 ± 0.14 °C; Supplementary Table S1). While four months (January, July, October, and December, Fig. 3a) were different in 2021 compared to 2020, as with DO, there was no consistent directional change in surface temperature among months that were different between years. Conversely, in 2021, deep water temperature was cooler in 2021 compared to 2020 (*p* < 0.001, annual mean ± SE, 2020 = 25.67 ± 0.13 °C, 2021 = 24.94 ± 0.10 °C; Supplementary Table S1). However, as with surface temperatures, the seven months in which deep water temperature differed between 2021 and 2020 (March, May–August, October, and December; Fig. 3a) were both cooler and warmer than the previous year (Supplementary Table S1, Fig. 3b).

**Figure 4:**
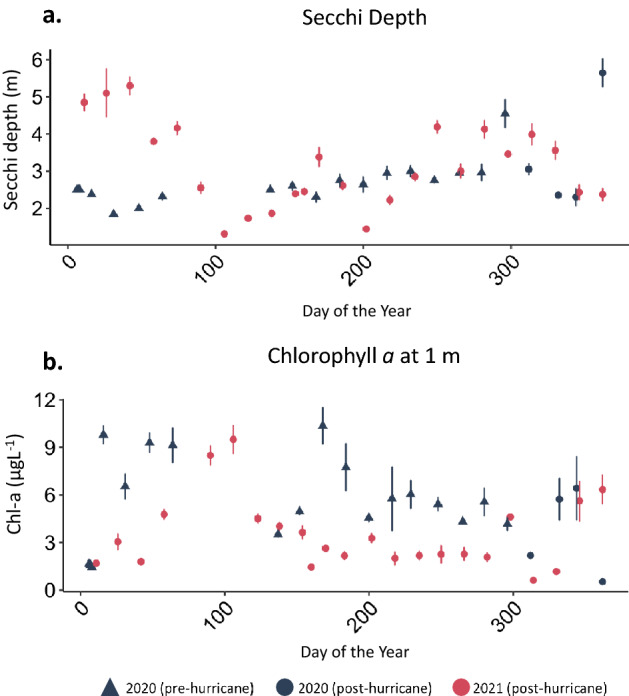
**(a**) Secchi depth and (**b**) Chl-a at five continuously monitored in-lake locations (mean ± SE).

Whereas thermo-physical structure appears to have only been minimally impacted, we observed pronounced changes in mean monthly Secchi depth and Chl-a between 2021 and 2020. Annual mean Secchi depth was only marginally greater in the year following the hurricanes compared to the year prior to the hurricanes (*p* = 0.04, annual mean ± SE, 2020 = 2.74 ± 0.08 m, 2021 = 3.14 ± 0.11 m; Supplementary Table S1). However, when comparing individual months, there was greater Secchi depth in December 2020 (*p* = 0.01, 2020 = 3.98 ± 0.59 m, 2021 = 2.40 ± 0.13 m), January 2021(*p* < 0.001, 2020 = 2.17 ± 0.06 m, 2021 = 4.97 ± 0.33 m) and February 2021 (*p* < 0.001, 2020 = 2.0 ± 0.0 m, 2021 = 4.55 ± 0.28 m) resulting in a clear period following Hurricanes Eta and Iota that was not present in the year preceding the hurricanes (Supplementary Table S1, Fig. 4a). This clear period was concurrent with (and driven by^19^) a decrease in Chl-a (measured at 1 m) in 2021 compared to 2020 (*p* < 0.001, annual mean ± SE, 2020 = 5.78 ± 0.28 µgL^−1^, 2021 = 3.51 ± 0.17 µgL^−1^; Supplementary Table S1). The difference in annual means was driven by differences in monthly means for the months directly following the hurricanes. Chl-a was lower in December 2020 (*p* = 0.08, 2020 = 3.47 ± 1.19 µgL^−1^, 2021 = 5.98 ± 0.78 µgL^−1^), January 2021 (*p* < 0.001, 2020 = 6.85 ± 0.72 µgL^−1^, 2021 = 2.36 ± 0.30 µgL^−1^), and February 2021 (*p* < 0.001, 2020 = 9.30 ± 0.62 µgL^−1^, 2021 = 3.28 ± 0.38 µgL^−1^) compared to the preceding year (Supplementary Table S1, Fig. 4b). Monthly Secchi depth and Chl-a were different in eight months (January, February, July to September, and November for both parameters in addition to May and December for Secchi depth and June and October for Chl-a). However, directional change was not always as expected, such as in July and August where Secchi depth was slightly (*p* = 0.01 and *p* = 0.02 for July and August, respectively) less in 2021 than in 2020 and Chl-a also decreased compared to 2020 levels (*p* < 0.001 and *p* = 0.001 for July and August, respectively; Supplementary Table S1).

**Figure 5:**
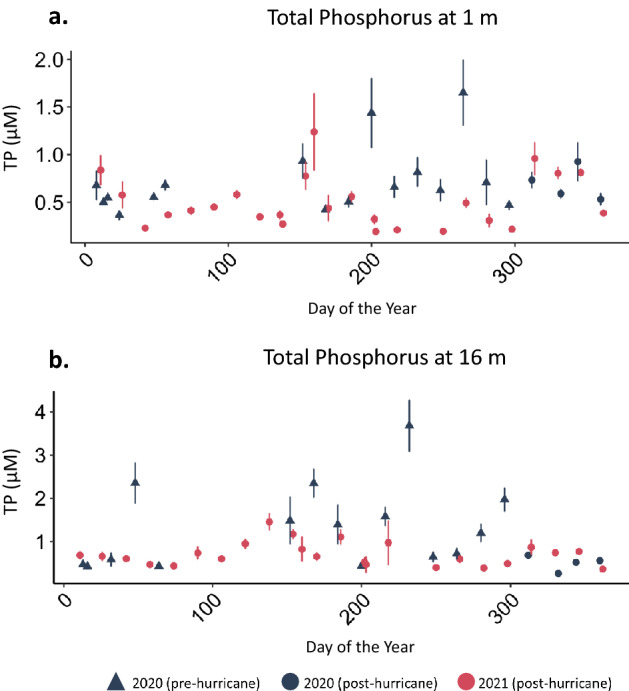
(**a**) Surface TP, and (**b**) deep TP at five continuously monitored in-lake locations (mean ± SE).

**Figure 6:**
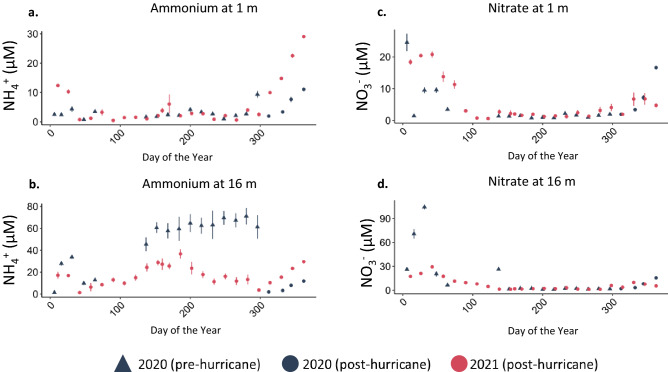
**(a**) Surface NH_4_^+^, (**b**) deep NH_4_^+^, (**c**) surface NO_3_^−^, and (**d**) deep NO_3_^−^ at five continuously monitored in-lake locations (mean ± SE).

**Figure 7:**
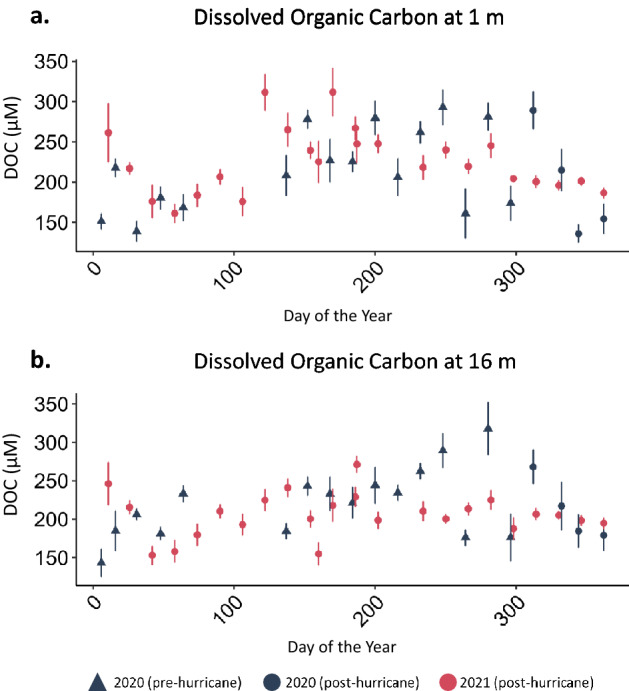
(**a**) Surface DOC, and (**b**) deep DOC at five continuously monitored in-lake locations (mean ± SE).

Similar to the differences in temperature in surface versus deep water, we observed a greater difference between 2020 and 2021 in deep compared to surface nutrient concentrations. Deep water DOC concentrations were lower in all months of 2021 compared to 2020 (*p* = 0.003, annual mean ± SE, 2020 = 224.27 ± 5.84 µM, 2021 = 206.50 ± 3.07 µM; Supplementary Table S1, Fig.3a). Similarly, we measured decreases in deep water TP (*p* < 0.001, annual mean ± SE, 2020 = 1.19 ± 0.09 µM, 2021 = 0.70 ± 0.03 µM; Supplementary Table S1, Fig. 5b) and NH_4_^+^ (*p* < 0.001, annual mean ± SE, 2020 = 42.90 ± 2.55 µM, 2021 = 17.05 ± 0.79 µM; Supplementary Table S1, Fig. 6b) between the 2 years. Hypolimnetic NH_4_^+^ was especially low during the stratified water column months (*p* < 0.001, annual mean ± SE, 2020 = 62.9 ± 2.52 µM, 2021 = 19.9 ± 1.22 µM; Supplementary Table S1, Figs. 3, 6b). NO_3_^−^ did not follow the same pattern as NH_4_^+^ (Figs. 3b, 6d). This decrease in the dissolved hypolimnetic C (Fig. 7) and N pool (Fig. 6) was accompanied by a corresponding decrease in the particulate C and N pool during stratified months with both particulate C and particulate N concentrations decreasing in the year following the hurricanes relative to the preceding year (particulate C during stratified months; *p* = 0.01, mean ± SE, 2020 = 71.8 ± 4.46 µM, 2021 = 55.2 ± 2.31 µM; particulate N during stratified months; *p* < 0.001, mean ± SE, 2020 = 15.6 ± 0.84 µM, 2021 = 11.4 ± 0.39 µM).

TP in surface waters was also less in 2021 compared to 2020 (*p* < 0.001, annual mean ± SE, 2020 = 0.72 ± 0.04 µM, 2021 = 0.48 ± 0.02 µM; Supplementary Table S1, Fig. 5a). In contrast, surface concentrations of NO_3_^−^ and NH_4_^+^ increased in 2021 relative to 2020. This differential increase in dissolved inorganic nitrogen (DIN) compared to TP resulted in large differences in stoichiometry between years. For example, in 2020, DIN:TP (atomic) was approximately 26 and 16 in January and February, respectively. In 2021, this ratio increased to approximately 43 and 62 in January and February, respectively.

While the observed increase in surface NO_3_^−^ was slight (*p* = 0.01, annual mean ± SE, 2020 = 4.20 ± 0.39 µM, 2021 = 5.69 ± 0.44 µM; Supplementary Table S1, Fig. 6c), we observed a much greater increase in surface NH_4_^+^ in 2021 relative to 2020 (*p* < 0.001, annual mean ± SE, 2020 = 3.67 ± 0.23 µM, 2021 = 6.16 ± 0.53 µM; Supplementary Table S1, Fig. 6a). The change in annual epilimnetic NH_4_^+^ was largely driven by water column concentrations following mixus the year following the hurricanes wherein NH_4_^+^ concentrations in November and December 2021 exceeded 2020 values (*p* < 0.001, Figs. 3, 5a). In 2020, mean surface NH_4_^+^ concentrations in November (2.62 ± 0.20 µM) and December (9.35 ± 0.64 µM) were similar to those in 2018 (November = 5.31 ± 0.51 µM, December = 9.48 ± 2.5 µM). Mean surface NH_4_^+^ concentrations were greater in November 2019 (17.08 ± 2.24 µM) and slightly less in December (4.10 ± 1.86 µM)^19^. In 2021, November mean surface NH_4_^+^ concentrations were within the range of previous years (12.4 ± 0.64 µM). However, in December 2021, surface NH_4_^+^ concentrations exceeded previously observed December conditions (25.8 ± 0.85 µM). The timing in peak surface NH_4_^+^ concentrations reflect, as in previous years, the timing of water column mixus.

Surface sediment samples collected in January preceding the November hurricanes were highly variably across the 16 sampling location. Sediments nearest the aquaculture operation were enriched in C and N (9.26 ± 0.97% C, 0.89 ± 0.09% N) compared to samples taken further from the net pens (5.76 ± 0.58% C, 0.53 ± 0.06% N). However, no other spatial patterns related to other nutrient sources (i.e., tributary loading) were identified. Collected sediments were high in organic matter (C = 6.20 ± 0.56%) and P (0.83 ± 0.09 mg g^−1^), and had a mean C:N (atomic) of 14.1 ± 1.02.

## Discussion

Extreme weather events, such as tropical cyclones, have been known to have wide and varying effects on lake ecosystems^14,28,29^. Here, we demonstrate that Hurricanes Eta and Iota had an ephemeral effect on Lake Yojoa and that Lake Yojoa’s trophic state was resilient to this perturbation. While there was an initial decrease in Secchi depth and increase in Chl-a in the first few weeks following the storms (Fig. 4), these changes were likely due to the supply of abundant hypolimnetic nutrients to the photic zone directly following mixus, as we have previously documented^19^, and not necessarily related to inputs from the hurricanes. Following this brief perennial algal bloom, Lake Yojoa experienced a clear water period with higher Secchi depth and lower Chl-a in January and February 2021 (two month mean ± SE, Secchi depth = 4.76 ± 0.21 m, Chl-a = 2.82 ± 0.25µgL^−1^; Fig. 4). This water column clearing was not observed in the year preceding the hurricane nor in observations made in Lake Yojoa in previous years^19^. The observed Chl-a decrease and corresponding Secchi depth increase could have been driven by numerable hurricane driven changes to systems dynamics, such as altered nutrient bioavailability. Alternatively, community structure may have been temporarily altered by the hurricane disturbance^13^.

The anomalous clear phase demonstrates how the combination of the two storms altered ecosystem function in Lake Yojoa. Furthermore, the reduction in hypolimnetic nutrient concentrations suggests that Hurricanes Eta and Iota, collectively, had a diluting rather than a nutrient loading effect on Lake Yojoa. This dilution effect is further supported by the observed decrease in hypolimnetic water temperatures after the onset of stratification (Figs. 2b, 3b), possibly due to the subduction of cooler contributing waters, and decreased hypolimnetic nutrient concentrations for NH_4_^+^, TP, and DOC (but not NO_3_^−^ which was low in both years, Figs. 6b,d, 5b, 7b). The decrease in hypolimnetic dissolved C and N during the stratified water column months may be partially explained by decreases in hypolimnetic particulate C and N in 2021 compared to 2020, resulting in reduced mineralization of organic matter during the stratified season.

Despite lower nutrient concentrations in the hypolimnion preceding water column turnover in November 2021, concentrations of NH_4_^+^, TP and DOC in surface waters following water column mixus were equal to or in excess of observed values in November 2020 (Figs. 6a, 5a, 7a). This pattern suggests that Lake Yojoa was resilient to change and able to maintain its trophic state even after a major dilution event. We suggest that this pulse of nutrients to the epilimnion during mixus may be due to an enhanced efflux of sediment nutrients that was coincident with mixus, and perhaps enhanced by the lower than normal water column nutrient concentrations in 2021.

In tropical lakes, biological processes define nutrient cycling, compared to in temperate lakes where physical processes define nutrient cycling for many months of a year^31^. As such, the absence of temperature limitation in warm-water lentic systems can produce nutrient regeneration patterns not observed in temperate ecosystems (where the majority of limnologic research occurs). Therefore, differences in sediment mineralization rates throughout the year are driven largely by seasonal changes in sediment redox conditions^32^. Seasonal changes in redox further indirectly influence mineralization rates through P availability^33^ and related changes in pH^34^.

Observed sediment nutrient effluxes vary substantially across systems^35–39^, but are often important contributors to whole-lake biogeochemistry such as in Lake Malawi where NH_4_^+^ efflux (0.44 mmol m^−2^ d^−1^) contributes up to 29% of total N inputs to the water column^40^. Furthermore, Lake Yojoa may experience even higher sediment nutrient efflux in regions impacted by aquaculture, similar to what has been observed in Lake Kariba where sediments located near aquaculture cages have a 4–25 fold increase in NH_4_^+^ and PO_4_^−3^ efflux^41^ compared to unimpacted sediments. Compared to Lake Kariba’s sediments (2.8–4.4% C, 0.26–0.49% N), Lake Yojoa’s aquaculture impacted sediments are even more enriched in C and N (9.26 ± 0.97% C, 0.89 ± 0.09% N). In addition, despite Lake Yojoa’s mesotrophic state, N and P in surface sediments were comparable to hypereutrophic lakes^42–44^, showing that substantial amounts of both N and P could be derived from sediments. For example, Lake Apopka, USA (with similar TP concentrations in surface sediments as Lake Yojoa, ~ 1.0 mg g^−1^), is a shallow system known to maintain Chl-a values above 100 µg L^−1^ (compared < 10 µg L^−1^ in Lake Yojoa) which are supported by sediment mixing and internal loading despite management of external nutrient inputs^44,45^. A system more comparable to Lake Yojoa in depth, location, and origin, Lake Amatitlán, Guatemala (max depth =  ~ 33) is hypereutrophic, but has similar surface sediment N (~ 0.63%) and TP (1.17 mg g^−1^) to Lake Yojoa^43^. Therefore, despite Lake Yojoa’s mesotrophic state, the present sediment nutrient content suggests that continued sediment nutrient pool enrichment may further promote the trophic state changes observed in the last forty years and be a key reason why Lake Yojoa’s trophic state was resilient to this major dilution event.

One possible explanation for the increased efflux of NH_4_^+^ in 2021 (compared to 2020 despite dissolved nutrient concentrations in the hypolimnion being lower in 2021 compared to 2020 preceding turnover) is defined by Fick’s first law^46^. Previous studies have used the concentration across the sediment water interface to estimate the release of nutrients from sediments to the overlying water^47,48^. Following this same principle, we can infer that compared to 2020, when overlying waters had a higher nutrient concentration, water column mixus in 2021 would have initiated a greater nutrient flux from Lake Yojoa’s sediments due to a larger concentration gradient between the water column and the surface sediment pool. Not only were overlying water nutrient concentrations diminished prior to mixing in 2021, but water column mixus would have further increased this concentration gradient due to the dilution effect of nutrient deplete epilimnetic water. This gradient may have resulted in an increased efflux of N and P from the sediments in 2021 compared to 2020 and previous years, thus making up for the observed hypolimnetic water column deficit in 2021 compared to 2020. This increased efflux then resulted in comparable mixed water column N and P values between the two years. While hypoxic conditions during stratification would be favorable for sediment P and N (as NH_4_^+^) release, the dramatic change in nutrient concentration gradients following turnover may also favor diffusive sediment N effluxes. No complementary studies which examine this phenomenon in warm monomictic lake ecosystems are currently available. However, given the well-documented link between internal nutrient loading and eutrophication, and the absence of temperature limitation (which is present during fall turnover in temperate lakes), putative variations in sediment nutrient efflux should be investigated further in tropical ecosystems.

Beyond assessing the impact of large, late-season precipitation events on an inland tropical lake, Hurricanes Eta and Iota presented a unique opportunity to understand the impact of decreased hypolimnetic nutrient concentrations under consistent stratification phenology in Lake Yojoa. Given the timing of such anomalous precipitation events during expected annual turnover, the dilution event created by Hurricanes Eta and Iota offers a proxy for temporarily decreased nutrient loading. This natural experiment demonstrates that short-lived nutrient reduction may have only an ephemeral impact on trophic state. The recovery of epilimnetic nutrient concentrations following turnover, despite lower dissolved nutrient concentrations in the hypolimnion in the preceding months, highlights the importance of other often unmeasured nutrient sources such as sediment flux from legacy nutrient inputs, other kinds of annual autochthonous loading, and algal entrainment^34,49–52^. Our observations in Lake Yojoa are in line with a review of 35 case studies in which recovery of lakes spanning subtropical and temperate latitudes were monitored following nutrient loading reductions. Most lakes reached a new TP equilibrium in 5 to 10 years following nutrient reductions^53^. However, with some exceptions^54^, deep lakes saw an increase in total N concentrations in years following nutrient reduction, despite decreased N loading^53^. Therefore, not only should nutrient loads to Lake Yojoa be decreased, but long-term monitoring is needed to understand the lasting impacts of these management actions. Most importantly, management practices should not be abandoned if there is not an immediate improvement in Lake Yojoa’s trophic state.

## Conclusion

Hurricanes Eta and Iota had an ephemeral impact on the trophic state of Lake Yojoa, increasing Secchi depth and decreasing Chl-a in the three months following the storms. Despite the intensity and proximity of Hurricanes Eta and Iota, the Lake Yojoa ecosystem was not impacted by the hurricanes, at least in terms of trophic state, suggesting that Lake Yojoa may be resilient to similar future extreme weather events. Additional studies of other large, inland lakes will help to further identify which ecosystem characteristics, beyond sediment nutrients, provided Lake Yojoa’s resiliency to hurricane disturbance. Likewise, understanding similar disturbance responses in other lake ecosystems with gradually increasing trophic states may provide insights into the potential effectiveness of nutrient reduction strategies. Ultimately, many lakes will, under future climate scenarios, bear the weight of greater storm intensities interfaced with cultural eutrophication. A mechanistic understanding of these ecological stressors and the underlying characteristics that determine lake responses will help to maintain the ecosystem services that local communities depend upon.

## Supplementary Information

Supplementary Table S1.

## Data Availability

The datasets generated during and/or analyzed during the current study are available from the corresponding author on reasonable request.
