# Supplementary material for: Trophic state resilience to hurricane disturbance of Lake Yojoa, Honduras
Source: Sci Rep. 2023 Apr 7;13:5681. doi: 10.1038/s41598-023-32712-3 (PMC10080183; doi:10.1038/s41598-023-32712-3)
Supplement: Supplementary file 1 — Supplementary Table S1. [file 41598_2023_32712_MOESM1_ESM.pdf]

Supplementary Table S1. Monthly and annual mean ± SE of thermo-physical structure and nutrient parameters. Cell color denotes p-value of monthly comparison (2020 vs. 2021, ANOVA).

								stratified						
		January	February	March	April	**May**	**June**	**July**	**August**	**September**	**October**	November	December	**Annual mean**
Secchi depth (m)	2020	2.2 ± 0.1	2.0 ± 0.0	2.3 ± 0.1	NA	2.6 ± 0.1	2.3 ± 0.1	2.7 ± 0.1	3.0 ± 0.1	2.9 ± 0.1	3.8 ± 0.3	2.7 ± 0.1	4.0 ± 0.6	**2.7 ± 0.1**
	2021	5.0 ± 0.3	4.6 ± 0.3	3.4 ± 0.3	1.3 ± 0.1	1.8 ± 0.1	2.8 ± 0.2	2.0 ± 0.2	2.5 ± 0.1	3.6 ± 0.2	3.8 ± 0.2	3.8 ± 0.2	2.40 ± 0.1	**3.1 ± 0.1**
Chl-a (μgL^{-1})	2020	6.9 ± 0.7	9.3 ± 0.6	9.1 ± 1.1	NA	4.2 ± 0.2	10.4 ± 1.2	6.2 ± 0.8	5.9 ± 1.1	4.9 ± 0.3	4.9 ± 0.5	4.0 ± 0.8	3.5 ± 1.2	**5.8 ± 0.3**
	2021	2.4 ± 0.3	3.3 ± 0.4	8.5 ± 0.6	9.5 ± 0.9	4.3 ± 0.2	2.7 ± 0.2	2.7 ± 0.2	2.1 ± 0.3	2.3 ± 0.3	3.3 ± 0.3	0.9 ± 0.1	6.0 ± 0.8	**3.5 ± 0.2**
NH_4^+ at 1 m (μM)	2020	3.2 ± 0.4	0.7 ± 0.2	3.5 ± 0.4	NA	1.7 ± 0.3	2.4 ± 0.5	3.3 ± 0.5	3.0 ± 0.3	1.7 ± 0.2	5.9 ± 1.0	2.6 ± 0.2	9.4 ± 0.6	**3.7 ± 0.2**
	2021	11.3 ± 0.5	1.0 ± 0.2	1.8 ± 0.6	1.4 ± 0.2	1.3 ± 0.3	4.0 ± 1.3	2.5 ± 0.2	2.4 ± 0.3	1.5 ± 0.3	3.5 ± 0.5	12.4 ± 0.6	25.8 ± 0.9	**6.2 ± 0.5**
NH_4^+ at 16 m (μM)	2020	21.1 ± 3.8	9.8 ± 1.5	12.8 ± 1.2	NA	55.6 ± 4.1	57.6 ± 6.9	62.0 ± 6.8	62.8 ± 7.2	68.4 ± 4.4	66.8 ± 6.3	2.6 ± 0.2	9.9 ± 0.7	**42.9 ± 2.6**
	2021	17.0 ± 1.5	4.0 ± 2.0	10.8 ± 1.3	9.9 ± 1.9	19.6 ± 2.5	27.3 ± 1.8	30.1 ± 3.7	14.6 ± 2.2	14.0 ± 2.2	9.7 ± 3.0	12.9 ± 0.7	26.5 ± 0.8	**17.1 ± 0.8**
NO_3^- at 1 m (μM)	2020	9.3 ± 1.8	9.6 ± 0.8	3.5 ± 0.4	NA	1.3 ± 0.1	1.5 ± 0.2	0.9 ± 0.1	1.5 ± 0.2	1.4 ± 0.1	1.8 ± 0.2	2.7 ± 0.2	11.9 ± 1.2	**4.2 ± 0.4**
	2021	19.4 ± 0.5	17.3 ± 1.2	7.2 ± 1.2	0.8 ± 0.1	1.7 ± 0.4	1.9 ± 0.4	1.6 ± 0.2	1.3 ± 0.1	1.9 ± 0.4	3.7 ± 0.8	4.3 ± 0.2	5.8 ± 1.0	**5.7 ± 0.4**
NO_3^- at 16 m (μM)	2020	67.2 ± 8.8	20.4 ± 3.1	6.5 ± 0.7	NA	9.8 ± 3.2	2.3 ± 0.6	1.7 ± 0.8	1.6 ± 0.3	1.7 ± 0.1	1.7 ± 0.1	2.6 ± 0.2	11.5 ± 1.1	**10.1 ± 1.6**
	2021	19.2 ± 0.7	23.4 ± 2.1	10.5 ± 1.1	7.9 ± 0.9	3.0 ± 1.1	1.4 ± 0.2	2.1 ± 0.3	2.5 ± 0.4	1.3 ± 0.1	3.5 ± 0.9	6.7 ± 1.3	6.5 ± 0.9	**7.2 ± 0.5**
TP at 1 m (μM)	2020	0.5 ± 0.0	0.6 ± 0.0	NA	NA	0.9 ± 0.2	0.4 ± 0.0	1.0 ± 0.2	0.7 ± 0.1	1.1 ± 0.2	0.6 ± 0.1	0.7 ± 0.1	0.7 ± 0.1	**0.7 ± 0.0**
	2021	0.7 ± 0.1	0.3 ± 0.0	0.4 ± 0.0	0.6 ± 0.0	0.3 ± 0.0	0.8 ± 0.1	0.4 ± 0.0	0.2 ± 0.0	0.3 ± 0.0	0.3 ± 0.0	0.9 ± 0.1	0.6 ± 0.1	**0.5 ± 0.0**
TP at 16 m (μM)	2020	0.5 ± 0.0	1.5 ± 0.4	0.4 ± 0.0	NA	1.5 ± 0.5	2.4 ± 0.3	0.9 ± 0.2	2.6 ± 0.4	0.7 ± 0.1	1.6 ± 0.2	0.5 ± 0.1	0.5 ± 0.0	**1.2 ± 0.1**
	2021	0.7 ± 0.1	0.5 ± 0.1	0.6 ± 0.1	0.6 ± 0.1	1.2 ± 0.1	0.9 ± 0.1	0.7 ± 0.1	1.0 ± 0.5	0.5 ± 0.1	0.4 ± 0.0	0.8 ± 0.1	0.6 ± 0.1	**0.7 ± 0.0**
DOC at 1 m (μM)	2020	171.1 ± 10.3	180.5 ± 14.6	168.3 ± 16.6	NA	249.4 ± 14.9	226.9 ± 26.9	254.0 ± 13.9	234.1 ± 14.7	227.0 ± 23.9	230.5 ± 18.4	252.1 ± 19.2	145.2 ± 10.9	**214.1 ± 5.7**
	2021	239.2 ± 18.9	168.6 ± 11.7	195.2 ± 8.8	176.0 ± 17.9	288.4 ± 15.9	264.1 ± 15.2	253.7 ± 9.9	218.3 ± 15.3	229.8 ± 7.2	224.9 ± 8.9	198.3 ± 4.8	193.9 ± 4.5	**225.3 ± 4.2**
DOC at 16 m (μM)	2020	178.3 ± 12.3	181.3 ± 8.8	233.3 ± 11.1	NA	222.2 ± 11.5	233.2 ± 22.0	232.8 ± 15.5	249.3 ± 7.9	232.7 ± 17.8	247.1 ± 27.7	244.0 ± 19.4	182.0 ± 14.4	**224.3 ± 5.8**
	2021	230.9 ± 14.8	155.6 ± 9.3	194.3 ± 9.1	192.9 ± 14.0	233.0 ± 9.0	195.9 ± 10.4	233.0 ± 8.5	210.4 ± 12.9	207.1 ± 5.4	206.5 ± 10.3	206.0 ± 4.6	196.5 ± 5.0	**206.5 ± 3.1**
Temperature at 2 m (C)	2020	24.8 ± 0.2	25.3 ± 0.3	25.4 ± 0.1	NA	28.2 ± 0.1	27.5 ± 0.1	27.7 ± 0.1	28.1 ± 0.1	28.4 ± 0.0	27.8 ± 0.1	25.4 ± 0.1	24.3 ± 0.1	**26.7 ± 0.2**
	2021	24.2 ± 0.1	24.9 ± 0.1	25.7 ± 0.2	26.6 ± 0.1	28.1 ± 0.1	28.0 ± 0.3	28.1 ± 0.1	28.0 ± 0.1	28.4 ± 0.1	28.3 ± 0.0	25.6 ± 0.1	25.0 ± 0.1	**26.7 ± 0.1**
Temperature at 16 m (C)	2020	23.8 ± 0.1	23.9 ± 0.1	24.0 ± 0.0	NA	25.5 ± 0.2	26.0 ± 0.1	26.5 ± 0.2	26.9 ± 0.1	27.1 ± 0.2	27.1 ± 0.1	25.3 ± 0.1	24.3 ± 0.1	**25.7 ± 0.1**
	2021	23.8 ± 0.1	23.7 ± 0.2	24.2 ± 0.0	24.3 ± 0.0	24.3 ± 0.0	24.6 ± 0.1	25.2 ± 0.1	26.4 ± 0.1	26.5 ± 0.1	27.5 ± 0.1	25.5 ± 0.1	24.5 ± 0.1	**24.9 ± 0.1**
Dissolved Oxygen at 2 m (mgL^{-1})	2020	8.5 ± 0.6	7.2 ± 0.1	8.0 ± 0.2	NA	7.0 ± 0.2	7.1 ± 0.2	6.6 ± 0.2	6.8 ± 0.2	6.7 ± 0.2	5.5 ± 0.3	5.4 ± 0.3	5.0 ± 0.3	**6.5 ± 0.1**
	2021	6.0 ± 0.3	7.2 ± 0.2	7.7 ± 0.2	9.5 ± 0.2	8.0 ± 0.1	7.3 ± 0.1	6.9 ± 0.1	6.8 ± 0.1	6.5 ± 0.1	7.3 ± 0.2	2.3 ± 0.6	8.2 ± 0.2	**6.8 ± 0.2**
Dissolved Oxygen at 16 m (mgL^{-1})	2020	5.0 ± 0.5	1.6 ± 0.6	3.7 ± 0.6	NA	0.0 ± 0.0	0.4 ± 0.1	1.4 ± 0.8	1.1 ± 0.7	0.7 ± 0.7	1.0 ± 0.4	4.6 ± 0.4	4.9 ± 0.3	**2.4 ± 0.3**
	2021	4.7 ± 0.2	4.1 ± 0.4	3.2 ± 0.5	2.7 ± 0.4	0.0 ± 0.0	0.0 ± 0.0	0.0 ± 0.0	0.6 ± 0.3	0.1 ± 0.1	2.4 ± 0.9	2.2 ± 0.6	5.0 ± 0.3	**2.0 ± 0.2**

Significant Differenes (2020 vs. 2021): | 0.001 | 0.01 | 0.05 |
